# CCAAT/enhancer-binding protein-β functions as a negative regulator of Wnt/β-catenin signaling through activation of *AXIN1* gene expression

**DOI:** 10.1038/s41419-018-1072-1

**Published:** 2018-10-03

**Authors:** Seoyoung Park, Mi-Sun Lee, Jungsug Gwak, Tae-Ik Choi, Youngseok Lee, Bong Gun Ju, Cheol-Hee Kim, Sangtaek Oh

**Affiliations:** 1 BK21 PLUS Program, Department of Bio and Fermentation Convergence Technology, Kookmin University, Seoul, 02707 Republic of Korea; 2 Department of Biology, Chungnam National University, Daejeon, 34134 Republic of Korea; 3 Department of Life Science, Sogang University, Seoul, 04107 Republic of Korea

## Abstract

Axin1, a concentration-limiting component of the β-catenin destruction complex, negatively regulates the Wnt/β-catenin pathway. Axin1 concentration is reported to be regulated by proteasomal degradation; however, its transcriptional regulation has not yet been reported. Here, we demonstrated that CCAAT/enhancer-binding protein-β (C/EBP-β) activates axis inhibition protein 1 (*AXIN1*) gene expression, thereby attenuating Wnt/β-catenin signaling. C/EBP-β interacted with *cis*-regulatory element for C/EBP-β in the 5′-upstream sequences of the *AXIN1* gene and increased *AXIN1* promoter activity. Functional analysis using *Drosophil*a and zebrafish models established that C/EBP-β negatively regulates the Wnt/β-catenin pathway. Small-molecule-based up-regulation of C/EBP-β induces *AXIN1* gene expression and down-regulates the intracellular β-catenin level, thereby inhibiting hepatoma cell growth. Thus, our findings provide a unique mechanistic insight into the regulation of Axin homeostasis and present a novel strategy for the development of anticancer therapeutics targeting Wnt/β-catenin signaling.

## Introduction

Alteration of the axis inhibition protein 1 (*AXIN1*) regulates the Wnt/β-catenin pathway and subsequently plays important roles in embryogenesis and tumorigenesis^1^. In this pathway, the scaffold protein Axin1 interacts with several proteins including casein kinase 1, glycogen synthase kinase-3β (GSK-3β), adenomatous polyposis coli (APC), and β-catenin through separate domains (which constitute the β-catenin destruction complex) to coordinate the sequential phosphorylation of β-catenin at residues Ser45, Thr41, Ser37, and Ser33^2,3^. The occurrence of phosphorylation serves as a target for degradation by the ubiquitin-dependent proteasome pathway^4^. Axin1 is recruited to the phosphorylated low-density lipoprotein receptor-related protein 5/6 (LRP5/6) co-receptors after the binding of Wnt ligands (Wnt1, Wnt3a, and Wnt8) to the Frizzled receptor and LRP5/6^5^. This leads to negative regulation of GSK-3β in the β-catenin destruction complex and in turn stabilization of β-catenin^6^. Axin1 is present at lower concentrations in the β-catenin destruction complex compared with the other components, and therefore acts as a concentration-limiting factor for assembly of this complex^7^. Thus, the levels of Axin1 are the key regulators in the Wnt/β-catenin pathway.

CCAAT/enhancer-binding protein-β (C/EBP-β), also known as NF-IL6 (nuclear factor for interleukin-6) or TCF5 (transcription factor 5), is a member of C/EBP family of transcription factors^8^. C/EPB-β is an intronless gene, the transcript of which encodes the in-frame translational products LAP1 (liver-enriched transcriptional activating protein 1 or LAP*), LAP2 (liver-enriched transcriptional activating protein 2 or LAP), and LIP (liver-enriched transcriptional inhibitory protein)^9^. These transcription factors regulate several classes of genes, such as proliferation/differentiation-related markers, metabolic enzymes, and cytokines^10^. In this study, we identify C/EBP-β as a bona fide transcriptional activator of the *AXIN1* gene expression. C/EBP-β promotes Axin1-mediated β-catenin degradation and thereby negatively regulates the Wnt/β-catenin pathway.

## Results

### *AXIN1* gene expression is activated during adipocyte differentiation

**Fig. 1:**
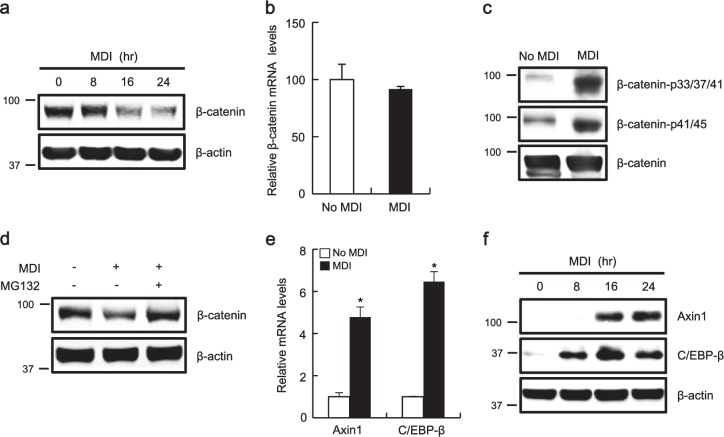
Activation of *AXIN1* gene expression during adipogenesis. **a** 3T3-L1 cell lysates were prepared at 8, 16, and 24 h after MDI treatment and subjected to western blot analysis using the indicated antibodies. **b** Real-time PCR for the analysis of β-catenin and β-actin was performed with total RNA prepared from 3T3-L1 cells treated with MDI for 24 h. **c** Cell lysates prepared from 3T3-L1 cells treated with MDI for 24 h were subjected to western blot analysis against the indicated antibodies. Identical amount of β-catenin was loaded in each lane. **d** Cytosolic proteins prepared from MDI-treated 3T3-L1 cells exposed to MG132 (10 μM) were subjected to western blot analysis against the indicated antibodies. **e** Real-time PCR for Axin1 and C/EBP-β expression was performed with total RNA prepared from 3T3-L1 cells treated with MDI for 24 h. In **b**, **e**, results are the average of three experiments, and bars indicate standard deviations. *P < 0.05, compared with the control. **f** Whole-cell lysates were prepared at 8, 16, and 24 h after MDI treatment and subjected to western blot analysis using the indicated antibodies

The Wnt/β-catenin pathway represses adipogenic differentiation of mesenchymal stem cells through the down-regulation of adipogenic transcription factors^11^. Inversely, we observed that adipogenic stimulation with MDI (3-isobutyl-1-methylxanthin, dexamethasone, and insulin) led to a reduction in the level of β-catenin without altering the β-catenin messenger RNA (mRNA) level (Fig. 1a, b), an increase of β-catenin phosphorylation at Ser33/37/Thr41/45 (Fig. 1c), and a decrease in the expression of *AXIN2* (Supplementary Fig. 1), a β-catenin-dependent gene, in 3T3-L1 preadipocytes. In addition, MDI-induced β-catenin down-regulation was nullified by the addition of MG132, a proteasome inhibitor (Fig. 1d), suggesting that β-catenin protein stability is negatively regulated during adipocyte differentiation. As β-catenin protein stability is mainly regulated by the β-catenin destruction complex, 3T3-L1 cells were incubated with the adipogenic inducers MDI, following which the mRNA levels of factors in this complex were measured by real-time polymerase chain reaction (PCR) to investigate the underlying mechanism of β-catenin degradation induced by adipogenic stimulation. As shown in Fig. 1e, the mRNA level of Axin1 was significantly up-regulated in response to treatment with MDI. Similar to mRNA expression, the protein concentrations of Axin1 also increased during MDI-induced adipocyte differentiation (Fig. 1f). In contrast, the mRNA and protein levels of other factors in the destruction complex were not altered by treatment with MDI (Supplementary Fig. 2). Under these conditions, the expression of C/EBP-β, a master transcription factor of adipogenesis, was consistently up-regulated (Fig. 1e, f), suggesting that *AXIN1* gene expression is activated in response to adipogenic stimulation.

### C/EBP-β is a bona fide transcriptional activator of *AXIN1* gene expression

**Fig. 2:**
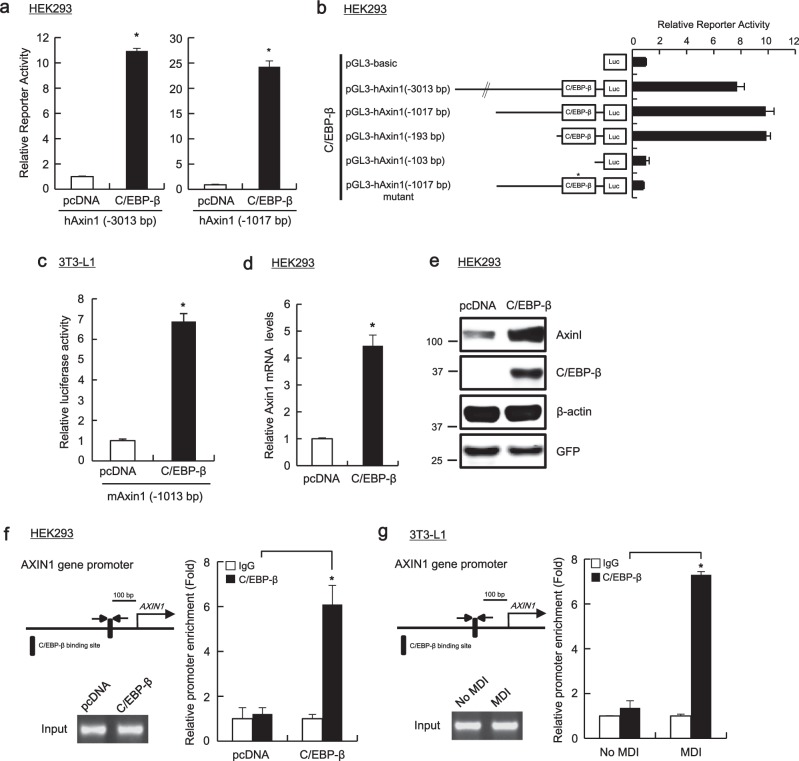
Identification of C/EBP-β as a bona fide transcriptional activator of *AXIN1* gene expression. **a**, **b** HEK293 cells were transfected with indicated Axin1 promoter constructs, C/EBP-β expression plasmid and pRL-CMV plasmid for 48 h. Luciferase activities were measured 48 h after transfection using the Dual Luciferase Reporter Assay System (Promega). TOPFlash activity is reported as relative light units (RLUs) normalized to Renilla luciferase activity. **c** 3T3-L1 cells were transfected with mAxin-1013 promoter constructs and C/EBP-β expression plasmid and pRL-CMV plasmid for 48 h. **d**–**f** HEK293 cells were transfected with pcDNA or C/EBP-β expression plasmids for 48 h. Total RNA was subjected to real-time PCR for Axin1 expression (**d**), and whole-cell lysates were subjected to western blot analysis and probed with the indicated antibodies (**e**). Chromatin samples were prepared from HEK293 cells and subjected to ChIP analysis with anti-C/EBP-β antibody or control IgG antibody. The amounts of immunoprecipitated C/EBP-β promoter regions were quantified by real-time PCR (**f**). **g** Chromatin samples were prepared from 3T3-L1 cells treated with MDI for 24 h and subjected to ChIP analysis with anti-C/EBP-β antibody or control IgG antibody. In **a–d**, **f** and **g**, results are the average of three experiments, and bars indicate standard deviations. *P < 0.05, compared with the control

To identify regulatory-interacting elements underlying *AXIN1* gene expression, we used the TFSEARCH method^12^ and analyzed a set of 5′-upstream sequences (1 kb from the ATG start codon) of *AXINI* across various species for the presence of shared motifs in their regulatory sequences. Accordingly, *cis*-regulatory elements that were conserved in human, mouse, rat, and zebrafish were identified. The most enriched *cis*-regulatory element was identified for C/EBP-β (liver-enriched transcriptional activating protein, LAP), a bZIP transcription factor (Supplementary Fig. 3). We further analyzed the effect of C/EBP-β on Axin1 promoter activity in human embryonic kidney 293 (HEK293) cells, which contains intact components of the Wnt/β-catenin pathway^13^. To this end, luciferase activity was measured after co-transfection of HEK293 cells with a C/EBP-β expression plasmid and an indicated reporter construct (hAxin1-1017 and hAxin1-3013). As shown in Fig. 2a, ectopic expression of C/EBP-β led to a dramatic activation of the reporter gene expression from hAxin1 promoter. Furthermore, while C/EBP-β led to the up-regulation of the hAxin1-193 promoter activity, the activity of hAxin1-103, which lacks C/EBP-β-binding motif, was not significantly affected. Alteration of the wild-type C/EBP-β-binding sequences (−119/−106; ACCTTTCCTAATCC) into the mutant-type (ACCGGTCCTAATCC) blocked the activation of the hAxin1 promoter in the presence of ectopic C/EBP-β expression (Fig. 2b). Overexpression of C/EBP-α or the LIP, a truncated isoform of C/EBP-β lacking the transactivation domain, showed no effect on promoter activity of hAxin1 (Supplementary Fig. 4). Moreover, ectopic expression of C/EPB-β led to a dramatic activation of the reporter gene expression from mouse Axin1 promoter in 3T3-L1 murine preadipocytes (Fig. 2c). We then analyzed the level of Axin1 endogenous mRNA and protein expression in the presence of C/EBP-β using real-time PCR and western blot analysis, respectively. Consistent with the reporter assay, C/EBP-β expression led to increased Axin1 mRNA and protein levels in HEK293 cells (Fig. 2d, e). Further, we used chromatin immunoprecipitation (ChIP) assays to examine whether C/EPB-β binds directly to the Axin1 promoter. As show in Fig. 2f, ectopic expression of C/EBP-β resulted in an increase in hAxin1 promoter occupancy by C/EBP-β in HEK293 cells. Additionally, association of C/EBP-β to the Axin1 promoter region containing the C/EBP-β-binding element was promoted by treatment with MDI (Fig. 2g). In contrast, we did not observe a significant enrichment of C/EBP-β on the glyceraldehyde 3-phosphate dehydrogenase (GAPDH) promoter (Supplementary Fig. 5a, b). Taken together, these results suggest C/EBP-β to be a bona fide transcriptional activator of *AXIN1* gene expression.

### C/EBP-β suppresses Wnt/β-catenin signaling by activating *AXIN1* gene expression

**Fig. 3:**
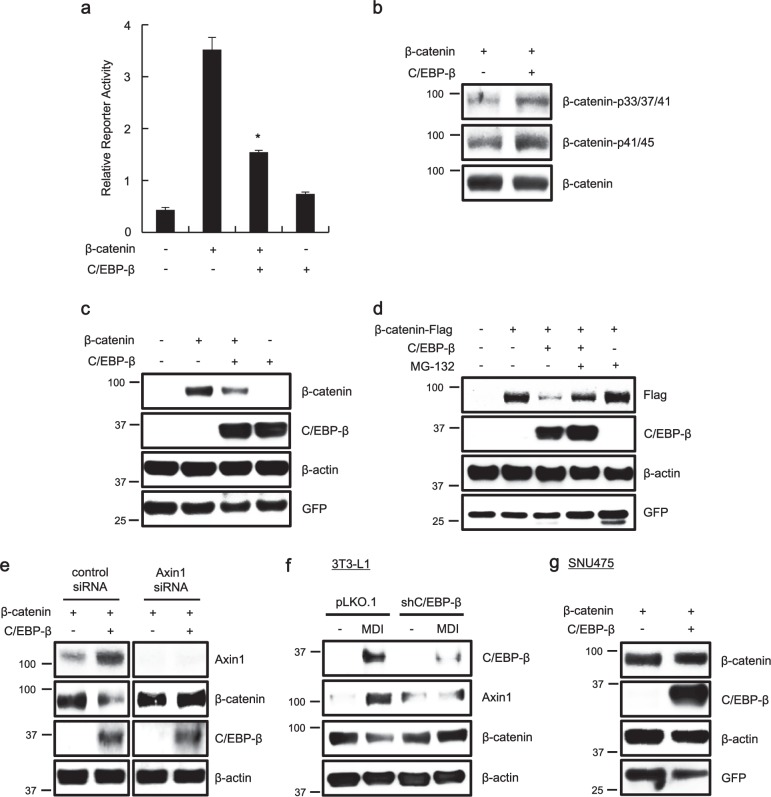
C/EBP-β-mediated β-catenin degradation is dependent on Axin1. **a** HEK293-FL reporter cells were transfected with β-catenin and C/EBP-β expression plasmids. Luciferase activities were measured 48 h after transfection using the Luciferase Reporter Assay System (Promega). TOPFlash activity was normalized with Celltiter-Glo (Promega) activity. Results are the average of three experiments, and bars indicate standard deviations. *P < 0.05, compared with the β-catenin-transfected control. **b** Cell lysates prepared from HEK293 cells transfected with β-catenin and C/EBP-β expression plasmids for 48 h were subjected to western blot analysis against the indicated antibodies. Identical amount of β-catenin was loaded in each lane. **c**, **d** Cytosolic proteins prepared from HEK293 cells transfected with β-catenin and C/EBP-β expression plasmids were subjected to western blot analysis against the indicated antibodies. Cells were exposed to MG132 (10 μM) for 8 h (**d**). **e** β-Catenin and C/EBP-β-expressing HEK293 cells were transfected with negative control (NC) siRNA (40 nM) or Axin1 siRNA (40 nM), following which cytosolic proteins were subjected to western blot analysis against the indicated antibodies. **f** 3T3-L1 cells were infected with pLKO.1 or shC/EBP-β lentivirus (Sigma-Aldrich), following which whole-cell lysates were subjected to western blot analysis against the indicated antibodies. **g** SNU475 cells were transfected with β-catenin and C/EBP-β expression plasmids were subjected to western blot analysis against the indicated antibodies

Axin1 is known to facilitate GSK-3β-mediated β-catenin phosphorylation and subsequently promote β-catenin degradation, thereby negatively regulating the Wnt/β-catenin pathway^2–4^. Since C/EBP-β leads to up-regulation of *AXIN1* gene expression, we next examined the effect of C/EBP-β on the Wnt/β-catenin pathway using HEK293-FL reporter cells, stably harboring a synthetic β-catenin/Tcf-dependent firefly luciferase (FL) reporter plasmid. β-Catenin response transcription that had increased due to β-catenin expression was down-regulated upon transfection of HEK293-FL reporter cells with the C/EBP-β expression plasmid (Fig. 3a). As expected, western blot analysis showed that the ectopic expression of C/EBP-β promoted phosphorylation of β-catenin Ser33/37/Thr41/Ser45 (Fig. 3b) and reduced the level of β-catenin protein in HEK293 cells (Fig. 3c). However, addition of the proteasomal inhibitor MG132 abolished C/EBP-β-mediated β-catenin down-regulation (Fig. 3d). Consistently, β-catenin ubiquitination was increased by C/EBP-β expression (Supplementary Fig. 6). Moreover, C/EBP-β expression was not able to induce β-catenin degradation upon small interfering RNA (siRNA)-mediated depletion of endogenous Axin1 in HEK293 cells (Fig. 3e). When we depleted endogenous C/EBP-β using short hairpin RNA (shRNA) lentivirus specific for C/EBP-β, adipogenic stimuli were not able to induce Axin1 expression and to down-regulate the level of β-catenin in 3T3-L1 cells (Fig. 3f). Notably, the level of β-catenin remained unchanged in *AXIN1* null-mutant SNU475 cells after expression of C/EPB-β (Fig. 3g). Taken together, these results indicate that C/EBP-β reduces intracellular β-catenin levels by activation of *AXIN1* gene expression.

### *c/ebp-β* acts a negative regulator of Wnt/β-catenin signaling in *Drosophila* and zebrafish

**Fig. 4:**
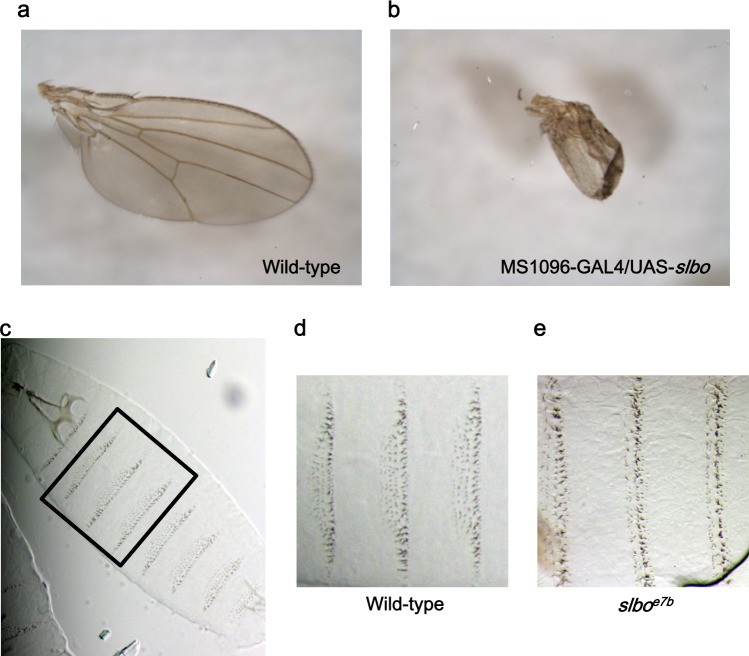
*slbo* negatively regulates Wg signaling in *Drosophila*. **a**, **b** Representative wing phenotypes of wild-type (**a**), MS1096-*GAL4*/*USA*-*slbo* (**b**). **c–e** *Drosophila* embryo cuticle prepared from wild-type and *slbo*^*e7b*^. Cuticle pattern of wild-type embryos (**c**). Representative phenotypes of wild-type (**d**) and *slbo*^*e7b*^ (**e**) in ventral abdominal segments 2–4

As Wingless (Wg), the *Drosophila* Wnt ortholog, has a well-established role in formation of the adult wing(s) and patterning the embryonic epidermis^14^, we investigated the function of C/EBP-β as a negative regulator of the Wnt/β-catenin pathway in *Drosophila*. To this end, we used GAL4/UAS system to overexpress *slbo*, a *Drosophila* ortholog of C/EPB-β, in fly wings and compared the resulting phenotypes to known effects of Wg mutations. As shown in Fig. 4a, b, wing-specific expression of *slbo* under the control of MS1096-GAL4 led to a reduction in the size of the wing, mimicking the phenotype of Wg loss-of-function mutation. Since epidermal cells with active Wg signaling secrete naked cuticle, we examined the effect of *slbo* on the Wg pathway in *slbo*^null^ mutant flies. Compared to wild-type flies, elimination of *slbo* gene resulted in the expansion of Wnt signaling as evidenced by increasing the secretion of naked cuticle (Fig. 4c–e), suggesting that *slbo* is a negative regulator of Wg signaling in *Drosophila*.

**Fig. 5:**
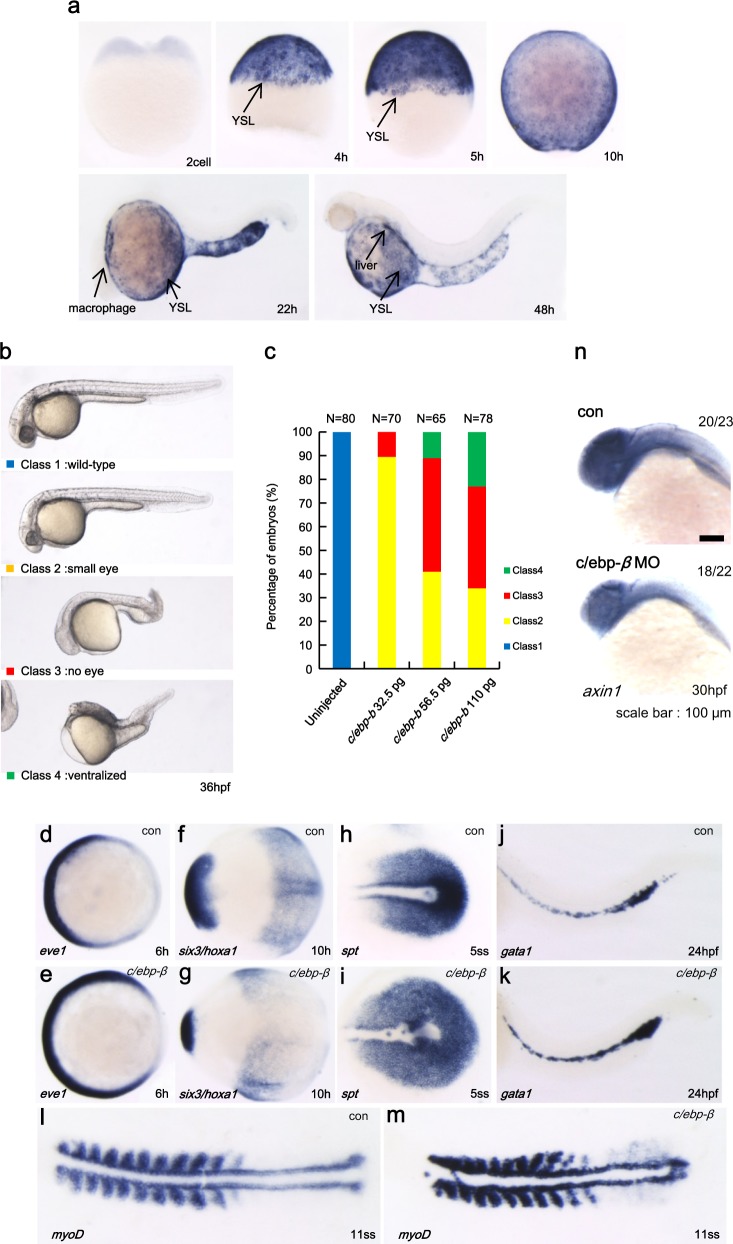
*c/ebp-β* negatively regulates Wnt signaling in zebrafish. **a** Spatial and temporal expression of *c/ebp-β* in developing zebrafish embryos determined by whole-mount in situ hybridization. **b** Injection of zebrafish *c/ebp-β* mRNA induced ventralized phenotype at 36 hpf embryos. **c** Percentage of embryos displaying specific phenotypes following *c/ebp-β* mRNA injection. **d–m** Expression of marker genes in *c/ebp-β* injected embryos. *eve1*, a ventral marker (**d**, **e**); *six3*, anterior neural marker; *hoxa1*, posterior neural marker at the tail bud stage (**f**, **g**); *spt*, posterior mesoderm marker (**h**, **i**) in intermediate cell mass (ICM) in the 5-somite stage embryo; gata1 expression in ICM (**j**, **k**); *myoD* expression in the 11-somite stage embryo (**l**, **m**). Embryos are shown in a dorsal view with anterior to the left. cont control mRNA, 5ss 5-somatic stage, 11ss 11-somatic stage. **n** The expression of *axin1* was examined by whole-mount in situ hybridization analysis after *c/ebp-β* MO injection. con control MO

We next tested whether C/EBP-β negatively regulates the Wnt/β-catenin pathway in zebrafish. To this end, we first analyzed the distribution of *c/ebp-β* transcripts by using whole-mount in situ hybridization starting from the two-cell stage to 48 h post fertilization (hpf). *c/ebp-β* is maternally provided and expressed ubiquitously throughout early development with higher levels being observed in the yolk syncytial layer and the liver (Fig. 5a). Further, we observed the effect of mRNA microinjection-mediated ectopic expression of zebrafish *c/ebp-β* on embryonic development. As shown in Fig. 5b, c, abnormal embryo populations that displayed ventralized phenotypes with small head/eye and expansion of intermediate cell mass (ICM) region, which is known to be derived from Axin1-mediated destabilization of β-catenin^15^, were observed in the *c/ebp-β* mRNA-overexpressing embryos at 36 hpf. We then examined the expression of several markers at multiple time points from 6 hpf through somitogenesis using whole-mount in situ hybridization analysis. The expression of *eve1*, a ventral marker, was increased in *c/ebp-β*-overexpressing embryo (Fig. 5d, e). Additionally, overexpression of *c/ebp-β* led to reduction of *six3*, an anterior neural marker, but an increase of *hoax1*, posterior neural marker, at the tail bud stage (Fig. 5f, g). In case of *spt*, a posterior mesoderm marker, the overexpression of *c/ebp-β* led to an increase in *spt* expression domain in the 5-somite stage embryo (Fig. 5h, i). Moreover, the injection of *c/ebp-β* mRNA resulted in a *gata1*-expressing domain in ICM (Fig. 5j, k). *myoD* expression at the 11-somite stage was used to observe the defects in tail development in *c/ebp-β* overexpressing embryo (Fig. 5l, m). We also used C/EPB-β-translation-blocking morpholino (5′-GATCTTAACACCC GC CGGATTGCG-3′), which was used in previous studies^16^, to deplete C/EPB-β and then examined the expression of *axin1* by whole-mount in situ hybridization analysis. *axin1* expression was reduced in *c/ebp-β* MO-injected embryos, compared to that of control embryos (Fig. 5n). The overall morphology of *c/ebp-β* MO-injected embryos was relatively normal (data not shown) because *c/ebp-β* MO may not be able to completely deplete endogenous C/EPB-β or functional isoforms. Taken together, these results indicate that C/EPB-β negatively regulates Wnt/β-catenin signaling in zebrafish and *Drosophila*.

### Small-molecule-based activation of C/EBP-β attenuates Wnt/β-catenin signaling via Axin1 up-regulation in hepatoma cells

**Fig. 6:**
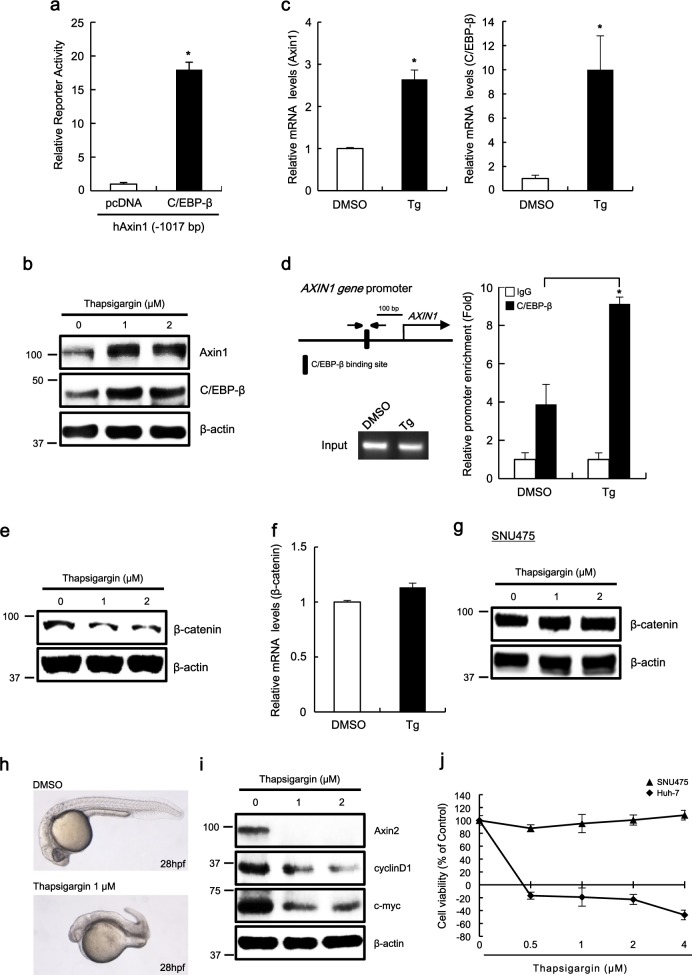
Characterization of a small-molecule activator of C/EBP-β expression in hepatoma. **a** Huh-7 cells were transfected with hAxin-1017 and C/EBP-β expression plasmid and pRL-CMV plasmid. After 48 h, luciferase activities were measured. **b–f** Huh-7 cells were incubated with the vehicle (DMSO) or thapsigargin (1 μM or indicated concentration) for 15 h. Whole-cell lysates prepared from Huh-7 cells were subjected to western blot analysis against the indicated antibodies (**b**). Real-time PCR for Axin1 and C/EBP-β were performed using total RNA prepared from Huh-7 cells (**c**). Chromatin samples were prepared and subsequently subjected to ChIP analysis against the anti-C/EBP-β antibody or control IgG. The amounts of immunoprecipitated C/EBP-β promoter regions were quantified by real-time PCR (**d**). Cytosolic proteins were subjected to western blot analysis against the β-catenin antibody (**e**) and total RNA was subjected to real-time PCR for β-catenin expression (**f**). **g** SNU475 cells were treated for 15 h with thapsigargin and cytosolic proteins were subsequently analyzed by western blot analysis against the β-catenin antibody. **h** Effect of thapsigargin on zebrafish embryonic development. **i** Lysates prepared from Huh-7 cells treated for 15 h with the vehicle (DMSO) or thapsigargin (1 μM) were subjected to western blot analysis against the indicated antibodies. **j** Effect of thapsigargin on hepatoma cell growth. In **a–c**, **d**, **f**, and **j**, results are the average of three experiments, and bars indicate standard deviations. *P < 0.05, compared with the control

We next used chemical biology approach to confirm C/EPB-β-mediated regulation of *AXIN1* gene expression and the Wnt/β-catenin pathway. Consistently, the overexpression of C/EPB-β, which controls hepatocyte differentiation and their characteristic quiescent state^17^, led to increased Axin1 promoter activity in Huh-7 hepatoma cells (Fig. 6a). Treatment of Huh-7 cells with thapsigargin, a known small-molecule activator of C/EBP-β^18^, increased the protein levels of both Axin1 and C/EBP-β (Fig. 6b), and their corresponding mRNA levels (Fig. 6c). However, these effects were not observed when we depleted C/EBP-β using shRNA lentivirus specific for C/EBP-β (Supplementary Fig. 7). ChIP assays showed that recruitment of C/EPB-β onto the Axin1 promoter increased in response to thapsigargin (Fig. 6d), while a significant enrichment of C/EBP-β was not found at the promoter region of GAPDH (Supplemental Fig. 5c). These results suggested that thapsigargin up-regulates *AXIN1* gene expression through the activation of C/EPB-β. We next examined the effects of thapsigargin on the level of intracellular β-catenin. As expected, incubation of Huh-7 cells with thapsigargin resulted in a decrease in the β-catenin levels without affecting its mRNA level (Fig. 6e, f). Notably, it did not affect the level of β-catenin in Axin1 null-mutant SNU475 cells (Fig. 6g), suggesting that it promotes Axin1-mediated β-catenin degradation. Consistently, the treatment of developing zebrafish embryos with thapsigargin induced ventralization (Fig. 6h). Further, we investigated the effect of thapsigargin on the expression of β-catenin downstream genes in Huh-7 cells. Incubation of thapsigargin with these cells led to a concentration-dependent decrease in the expression of well-established β-catenin-dependent genes such as cyclin D1, c-myc, and Axin2 (Fig. 6i)^19–21^. The specific reduction of β-catenin by antisense oligonucleotides or siRNA has been shown to inhibit the proliferation of cancer cells in vitro as well as tumor growth in a xenograft mouse model^22,23^. Therefore, we evaluated here whether thapsigargin inhibited the growth of hepatoma cells by incubating Huh-7 cells with its varying concentrations and monitoring subsequent cell growth. Thapsigargin suppressed the growth of these cells in a concentration-dependent manner without affecting SNU475 cell growth (Fig. 6j).

## Discussion

**Fig. 7:**
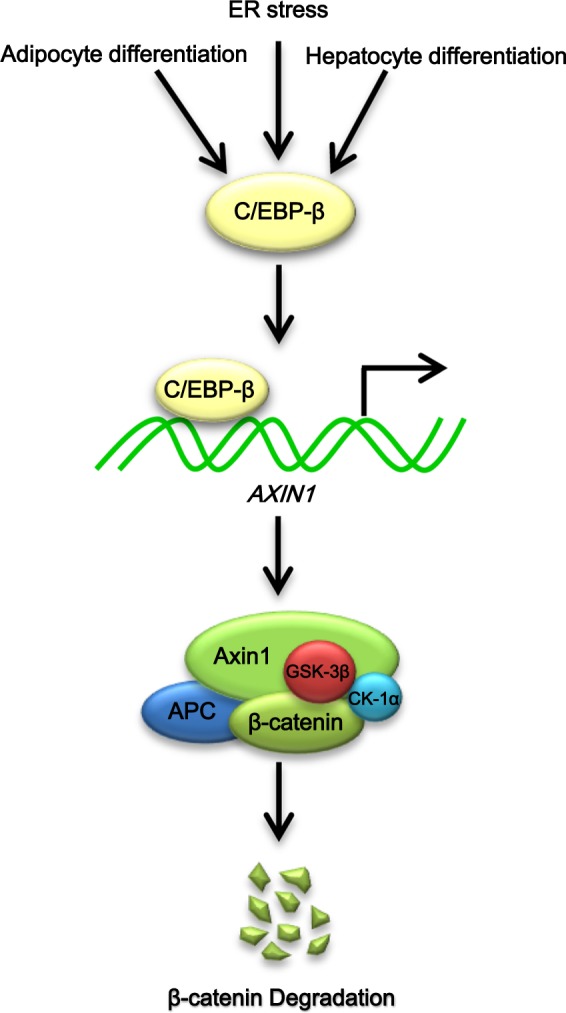
Schematic model for C/EBP-β mediated regulation of Axin1 homeostasis and Wnt signaling

The intracellular concentration of Axin1, a rate-limiting factor in β-catenin degradation, has been reported to be predominantly regulated by the ubiquitin-dependent proteasomal degradation pathway. Smurf2, a HECT-type E3 ubiquitin ligase, is known to promote Axin1 degradation^24^, while RNF-146, a RING-domain E3 ubiquitin ligase, is known to mediate tankyrase-dependent Axin1 degradation^25^. The current study elucidated a unique mechanism for regulating Axin1 concentration. The transcription factor C/EPB-β (LAP)-mediated activation of *AXIN1* expression, and subsequent regulation of Wnt/β-catenin signaling (Fig. 7) is a key finding of this study.

C/EBPβ exhibits anti-proliferative activity depending on specific cellular context^10^. C/EPB-β induces oncogene-induced senescence through a mechanism that requires RB/E2F repressor complexes^26^. During monocyte development, LIP appears to be associated with proliferation at the myelomonocytic progenitor stage, while LAP is associated with the inhibition of proliferation and expression of differentiation-related genes at later developmental stages^27^. Additionally, transformation of a granulocytic cell line by BCR/ABL is linked to the down-regulation of C/EPB-β expression, and its overexpression has been shown to inhibit proliferation of the BCR/ABL-transformed cells^28^. Furthermore, actively dividing hepatocytes are reported to specifically exclude the expression of C/EPB-β and the forced expression of C/EPB-β is shown to arrest the progression of cell cycle from the G_1_ phase to the S phase in hepatoma cells during postnatal development^17^. Systemic administration of C/EPB-β expression plasmid suppresses the growth of human colon cancer in nude mice^29^. However, the mechanism underlying the C/EPB-β-mediated inhibition of cell proliferation remains to be elucidated. In this study, we used a chemical biology approach to identify that activation of C/EPB-β with thapsigargin may inhibit hepatocyte proliferation through Axin1-mediated β-catenin degradation (Fig. 7). Taken together, our results suggest that the transcriptional activation of *AXIN1* gene by thapsigargin may provide a pharmacological basis for therapeutic intervention against Wnt-dependent cancers.

## Materials and methods

### Cell culture and differentiation

HEK293, SNU475, Huh-7, and 3T3-L1 cells were obtained from the American Type Culture Collection (Manassas, VA, USA). HEK293 was maintained in Dulbecco’s modified Eagle’s medium (DMEM) supplemented with 10% fetal bovine serum (FBS), 120 g/mL penicillin, and 200 g/mL streptomycin. 3T3-L1 cells were maintained in DMEM supplemented with 10% FCS, 120 g/mL penicillin, and 200 g/mL streptomycin. SNU475 and Huh-7 were maintained in Roswell Park Memorial Institute 1640 (RPMI 1640) supplemented with 10% FBS, 120 g/mL penicillin, and 200 g/mL streptomycin. HEK293-FL reporter (TOPFlash) and control (FOPFlash) cells were established as previously described^30^. 3T3-L1 preadipocytes were grown in DMEM supplemented with 10% FBS in order to induce adipogenesis. After 2 days of confluence (day 0), cells were incubated with 10% FBS, 0.5 mM of 3-isobutyl-1-methylxanthine, 1 μM dexamethasone, and 10 μg/mL insulin for 24 h.

### Plasmids, transfection, and luciferase assay

A bacterial artificial chromosome clone containing the region corresponding to Axin1 was used as a PCR template for cloning the promoter region of the human and mouse Axin1 genes. The amplified DNA fragments were sub-cloned into the luciferase reporter vector, pGL3-basic (Promega, Madison, WI, USA). Site-directed mutagenesis of the C/EBP-β-binding site was performed according to the QuikChange^TM^ Site-Directed Mutagenesis Kit (Agilent Technologies, Santa Clara, CA, USA). All clones and mutants were verified by sequencing. The pTOPflash reporter plasmid was obtained from Upstate Biotechnology (Lake Placid, NY, USA). pCMV-RL plasmid was purchased from Promega (Madison, Madison, WI, USA). Transfections were performed using Lipofectamine 2000 (Invitrogen, Carlsbad, CA, USA), according to the manufacturer’s instructions. Luciferase assay was performed using the Luciferase Assay System (Promega, Madison, WI, USA).

### Western blot analysis

Cytosolic fractions were prepared as previously described^30^. Proteins were separated by SDS-PAGE in a 4–12% gradient gel (Invitrogen, Carlsbad, CA, USA) and transferred to a nitrocellulose membrane (Bio-Rad, Hercules, CA, USA). The membrane was blocked with 5% nonfat milk and probed with anti-β-catenin (BD Transduction Laboratories, 610154, 1:1000), anti-phospho-β-catenin (Ser33/37/Thr41) (Cell Signaling Technology, #9561S, 1:1000), anti-phospho-β-catenin (Thr41/Ser45) (Cell Signaling Technology, #9565S, 1:1000), anti-Axin1 (Cell Signaling Technology, #2087S), anti-Axin2 (Cell Signaling Technology, #2151S, 1:1000), anti-C/EBPβ (Santa Cruz Biotechnology, sc-150, 1:500), anti-cyclinD1 (Santa Cruz Biotechnology, sc-20044, 1:500), anti-c-myc (Santa Cruz Biotechnology, sc-40, 1:500), anti-GFP (Invitrogen, A11122, 1:1000), and anti-actin (Sigma-Aldrich, A1978, 1:2000) antibodies. The membranes were then incubated with horseradish-peroxidase-conjugated anti-mouse IgG (Santa Cruz Biotechnology, sc-2004, 1:1000) or anti-rabbit IgG (Santa Cruz Biotechnology, sc-2031, 1:1000) and visualized using the ECL system (Santa Cruz Biotechnology, sc-2048).

### RNA extraction and real-time PCR

Total RNA was isolated using the TRIzol reagent (Invitrogen, Carlsbad, CA, USA) according to the manufacturer’s instructions. Complementary DNA (cDNA) synthesis, reverse transcription, and PCR were performed as previously described^31^ The amplified DNA was separated on 2% agarose gels and stained with ethidium bromide. For real-time PCR, the CFX96 qPCR system (Bio-Rad, Hercules, CA, USA) was used. Reactions were carried out in 96-well optical reaction plates in a 20 μl final volume containing 10 μl of the 2X SsoFast™ EvaGreen^®^ Supermixes (Bio-Rad, Hercules, CA, USA), 1 μl of each gene-specific forward primer, 1 μl of each gene-specific reverse primer, 1 μl of diluted cDNA sample, and 7 μl of water. After an initial denaturing step for 10 min at 95 °C, conditions for cycling were set to 40 cycles of 30 s at 94 °C, 30 s at 58 °C, and 30 s at 72 °C. The changes in gene expression were quantified by the comparative Ct method by calculating the relative fold changes normalized against β-actin expression.

### Small interfering RNA, short hairpin RNA lentivirus-mediated knockdown

Synthetic siRNAs targeting C/EBP-β were purchased from Applied Biosystems/Ambion (Carlsbad, CA, USA). Targeted sequences were as follows: C/EBP-β sense, 5′-GGCCCUGAGUAAUCGCUUAtt-3′ and antisense, 5′-UAAGCGAUUACUCAGGGCCcg-3′. AccuTarget™ Negative Control siRNA (Bioneer, Daejeon, Republic of Korea) was used as a control. shRNA lentivirus plasmids specific for C/EBP-β were purchased from Sigma-Aldrich (St. Louis, MO, USA)

### Chromatin immunoprecipitation

Cells were fixed with 1% formaldehyde for 10 min at room temperature. ChIP assays were performed using the Chromatin Immunoprecipitation (ChIP) Assay Kit (Upstate Biotechnology, Lake Placid, NY, USA) according to the manufacturer’s instructions. Chromatin extracts were immunoprecipitated with IgG (Santa Cruz Biotechnology, sc-69786, 2 μg) or anti-C/EBP-β antibodies (Santa Cruz Biotechnology, sc-150X, 2 μg). DNA-associated protein was analyzed with real-time PCR using primers targeting Axin1-binding site (−112/104). Primers used PCR were: forward, 5′-GGCCAAATATGTCTCTGCTGG-3′ and reverse, 5′-GCGCTGCACCCTAATACCTC-3′ (for mouse Axin1-binding site), and forward, 5′-GGGCATCCCTTCTTAGCATGAG-3′ and reverse, 5′-CGCTGCACCCTAATA CATCAGT AC-3′ (for human Axin1-binding site).

### Zebrafish maintenance

Zebrafish were maintained at 28.5 °C on a 14 h light/10 h dark cycle. Wild-type embryos were cultured in Ringer’s solution (116 mM NaCl, 2.9 mM KCl, 1.8 mM CaCl_2_, 5 mM HEPES, pH 7.2) and treated with PTU (phenylthiocarbamide, 1-phenyl-2-thiourea; Sigma) to suppress pigmentation. Embryonic stages were determined by the hpf and microscopic observation of gross morphology.

### Whole-mount in situ hybridization

To make an antisense RNA probe, the amplified zebrafish *c/ebp-β* PCR product was sub-cloned into the pGEM T-easy vector (Promega, Madison, WI, USA), linearized with *Nco*І, and transcribed in vitro by using SP6 polymerase and digoxigenin-labeled UTP (Roche). Whole-mount in situ hybridization was performed using standard protocols.

### Microinjection of *c/ebp-β* mRNA

Capped sense RNA encoding *c/ebp-β* was synthesized with SP6 RNA polymerase (Ambion, Austin, TX, USA) after linearization of pCS2 + *c/ebp-β*. The synthesized mRNA capped RNA was dissolved in nuclease-free water containing 0.2% phenol red as a tracking dye. Microinjections were performed in embryos at one-cell to four-cell embryos by using a microinjector (WPI).

### Cuticle preparation of *Drosophila* embryo

Put flies into egg-laying cages with apple juice agar plates and incubate at 25 °C in the dark until 50–100 eggs have been laid, and then allow embryos to age at 25 °C until before they hatch. After unhatched eggs were dechorionated with breach (8% sodium hypochlorite solution), rinse one more time with distilled water. Remove the vitelline membrane using hypodermic needle, and transfer to a drop of 1:1 lactic acid:Hoyer’s-based medium (Hoyer’s-based mountant) on a glass microscope slide. After that place a coverslip onto the slide, and then incubate the slide at approx. 60–65 °C overnight in an oven to allow digestion of internal tissues and clearing of the larvae. The control strain used in this study was *w*^*1118*^. We obtained *slbo*^*e7b*^ from the Bloomington Drosophila Stock Center (https://bdsc.indiana.edu/).

### Treatment of thapsigargin with developing zebrafish embryos

Wild-type embryos were treated with 1 μM of thapsigargin from the 50% epiboly stage to 28 hpf, while control embryos were treated with DMSO.

### Statistical analysis

Student’s *t* tests were used to compare means between control and experimental groups. All experiments were performed three times. Statistical significance was set at *P* < 0.05. Results are presented as the mean ± SD.

## Electronic supplementary material

Supplemental Figures
