# Supplementary material for: CCAAT/enhancer-binding protein-β functions as a negative regulator of Wnt/β-catenin signaling through activation of AXIN1 gene expression
Source: Cell Death Dis. 2018 Oct 3;9(10):1023. doi: 10.1038/s41419-018-1072-1 (PMC6170413; doi:10.1038/s41419-018-1072-1)
Supplement: Supplementary file 1 — Supplemental Figures [file 41419_2018_1072_MOESM1_ESM.pptx]

## Slide 1
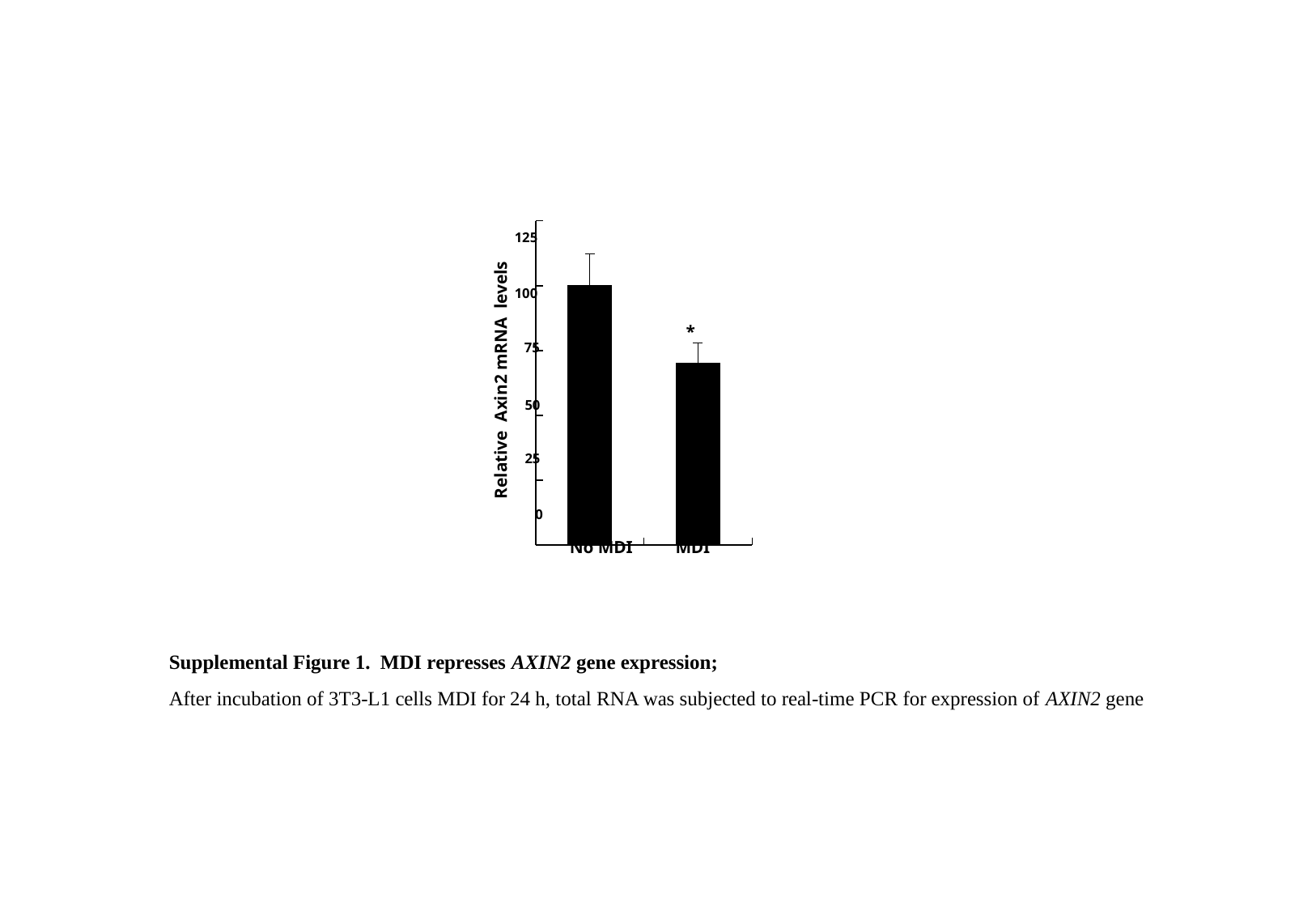

### Chart
| Category | |
|---|---|125
100
*
75
Relative Axin2 mRNA levels
50
25
0
MDI
No MDI
Supplemental Figure 1. MDI represses AXIN2 gene expression;
After incubation of 3T3-L1 cells MDI for 24 h, total RNA was subjected to real-time PCR for expression of AXIN2 gene

## Slide 2
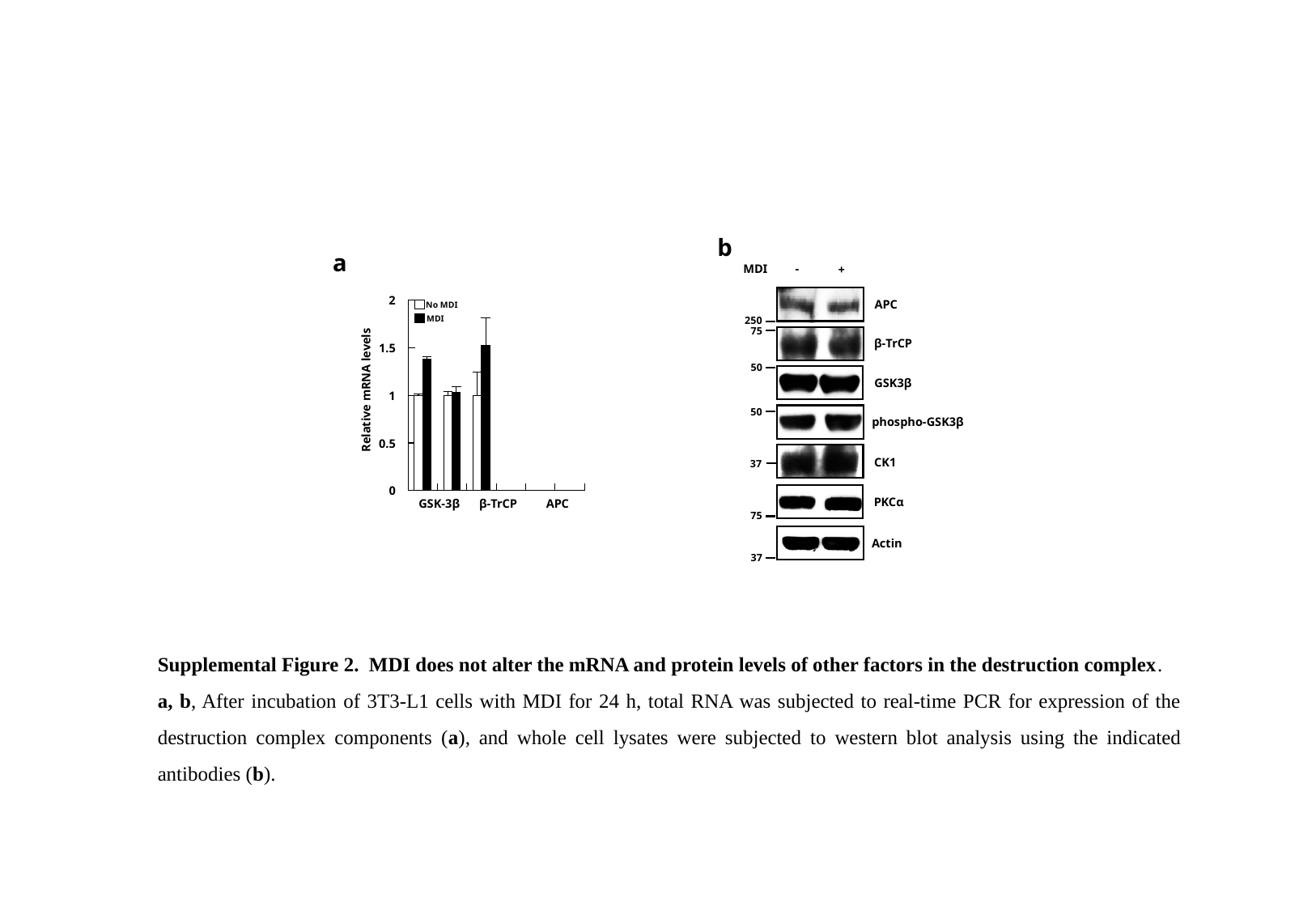

b
a
-
MDI
+
APC
β-TrCP
GSK3β
phospho-GSK3β
CK1
PKCα
Actin
2
[unsupported chart]
No MDI
MDI
1.5
Relative mRNA levels
1
0.5
0
GSK-3β
APC
β-TrCP
250
75
50
50
37
75
37
Supplemental Figure 2. MDI does not alter the mRNA and protein levels of other factors in the destruction complex.
a, b, After incubation of 3T3-L1 cells with MDI for 24 h, total RNA was subjected to real-time PCR for expression of the destruction complex components (a), and whole cell lysates were subjected to western blot analysis using the indicated antibodies (b).

## Slide 3
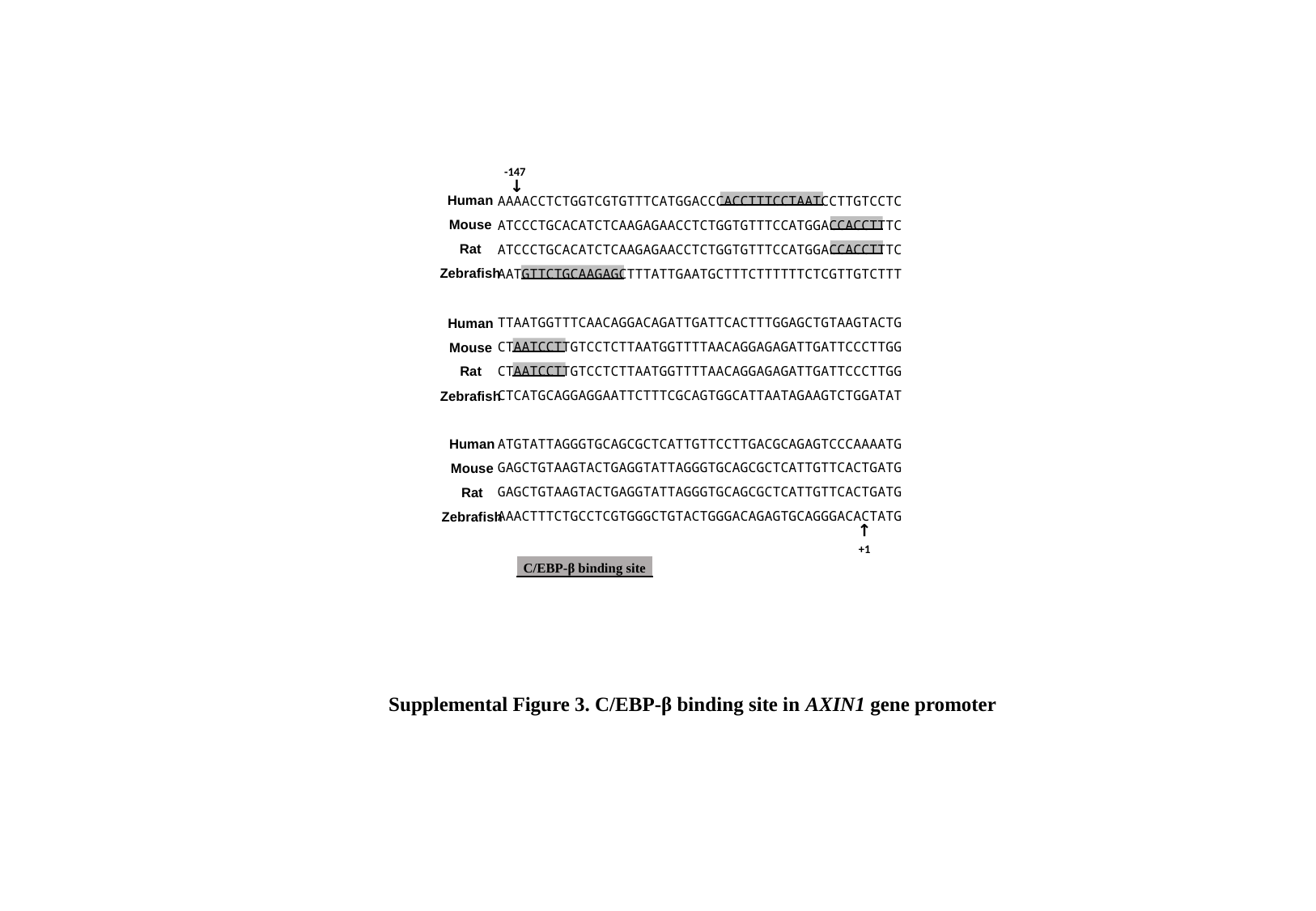

-147
↓
Human
Mouse
Rat
Zebrafish
AAAACCTCTGGTCGTGTTTCATGGACCCACCTTTCCTAATCCTTGTCCTC
ATCCCTGCACATCTCAAGAGAACCTCTGGTGTTTCCATGGACCACCTTTC
ATCCCTGCACATCTCAAGAGAACCTCTGGTGTTTCCATGGACCACCTTTC
AATGTTCTGCAAGAGCTTTATTGAATGCTTTCTTTTTTCTCGTTGTCTTT
TTAATGGTTTCAACAGGACAGATTGATTCACTTTGGAGCTGTAAGTACTG
CTAATCCTTGTCCTCTTAATGGTTTTAACAGGAGAGATTGATTCCCTTGG
CTAATCCTTGTCCTCTTAATGGTTTTAACAGGAGAGATTGATTCCCTTGG
CTCATGCAGGAGGAATTCTTTCGCAGTGGCATTAATAGAAGTCTGGATAT
ATGTATTAGGGTGCAGCGCTCATTGTTCCTTGACGCAGAGTCCCAAAATG
GAGCTGTAAGTACTGAGGTATTAGGGTGCAGCGCTCATTGTTCACTGATG
GAGCTGTAAGTACTGAGGTATTAGGGTGCAGCGCTCATTGTTCACTGATG
AAACTTTCTGCCTCGTGGGCTGTACTGGGACAGAGTGCAGGGACACTATG
Human
Mouse
Rat
Zebrafish
Human
Mouse
Rat
Zebrafish
↓
+1
C/EBP-β binding site
Supplemental Figure 3. C/EBP-β binding site in AXIN1 gene promoter

## Slide 4
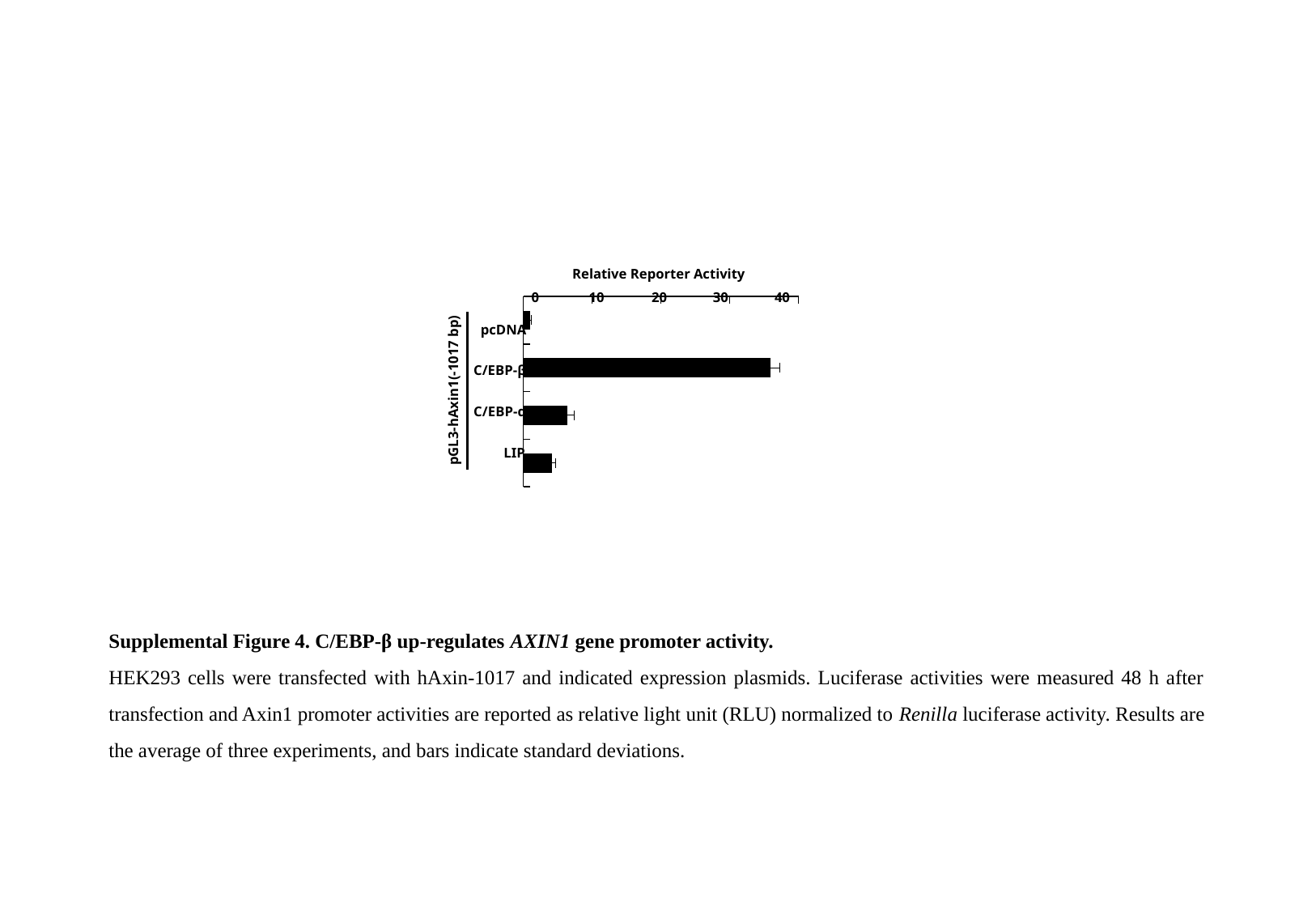

Relative Reporter Activity
0
10
20
30
40
pcDNA
C/EBP-β
pGL3-hAxin1(-1017 bp)
C/EBP-α
LIP
### Chart
| Category | |
|---|---|
| pcDNA | 1.0 |
| C/EBPβ | 35.93365767867211 |
| C/EBPα | 6.3414198102573485 |
| LIP | 4.148588938277644 |Supplemental Figure 4. C/EBP-β up-regulates AXIN1 gene promoter activity.
HEK293 cells were transfected with hAxin-1017 and indicated expression plasmids. Luciferase activities were measured 48 h after transfection and Axin1 promoter activities are reported as relative light unit (RLU) normalized to Renilla luciferase activity. Results are the average of three experiments, and bars indicate standard deviations.

## Slide 5
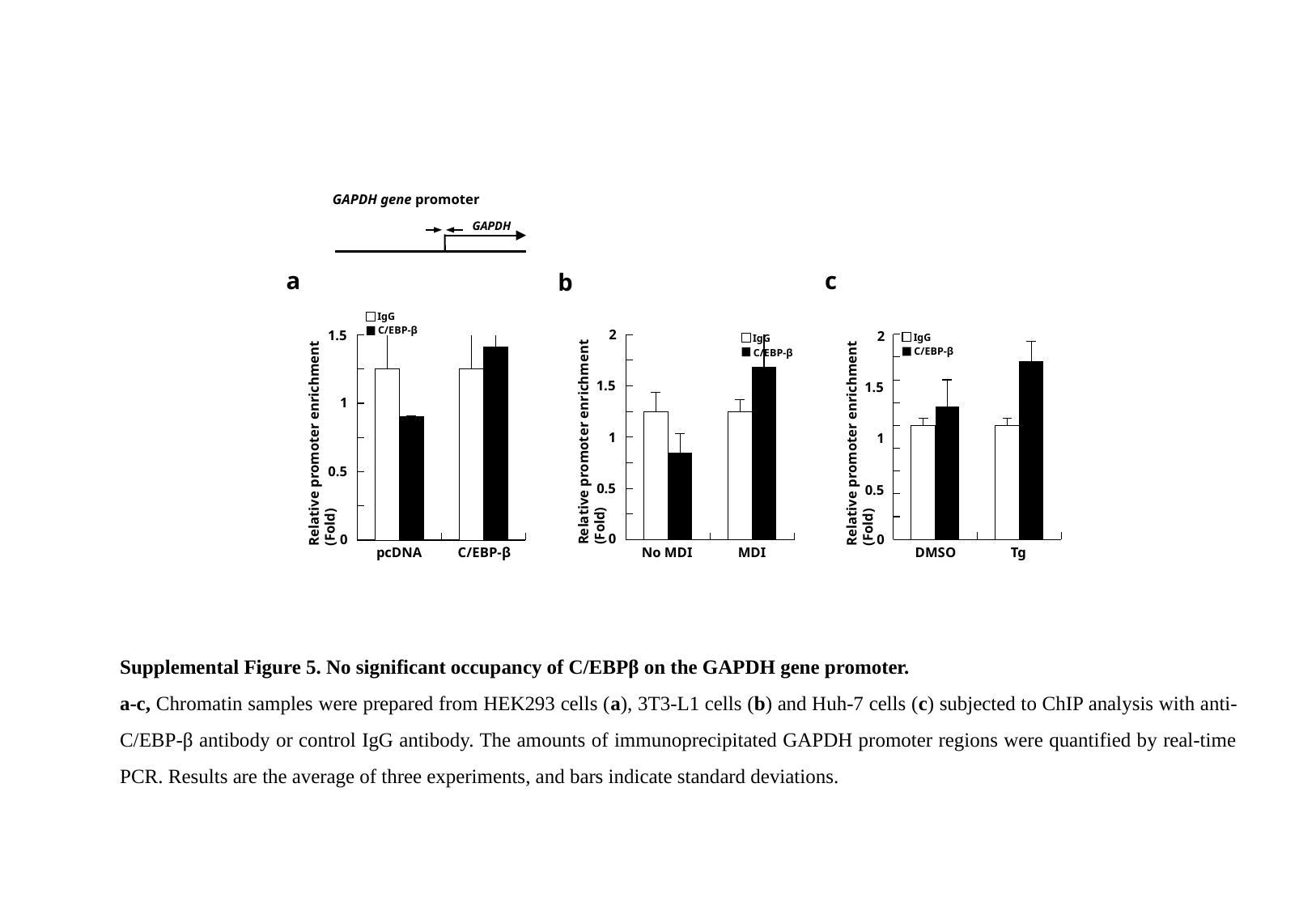

GAPDH gene promoter
GAPDH
c
a
b
IgG
C/EBP-β
### Chart
| Category | | |
|---|---|---|1.5
1
Relative promoter enrichment (Fold)
0.5
0
C/EBP-β
pcDNA
### Chart
| Category | | |
|---|---|---|2
1.5
1
Relative promoter enrichment (Fold)
0.5
0
IgG
C/EBP-β
Tg
DMSO
### Chart
| Category | | |
|---|---|---|2
1.5
1
Relative promoter enrichment (Fold)
0.5
0
IgG
C/EBP-β
No MDI
MDI
Supplemental Figure 5. No significant occupancy of C/EBPβ on the GAPDH gene promoter.
a-c, Chromatin samples were prepared from HEK293 cells (a), 3T3-L1 cells (b) and Huh-7 cells (c) subjected to ChIP analysis with anti-C/EBP-β antibody or control IgG antibody. The amounts of immunoprecipitated GAPDH promoter regions were quantified by real-time PCR. Results are the average of three experiments, and bars indicate standard deviations.

## Slide 6
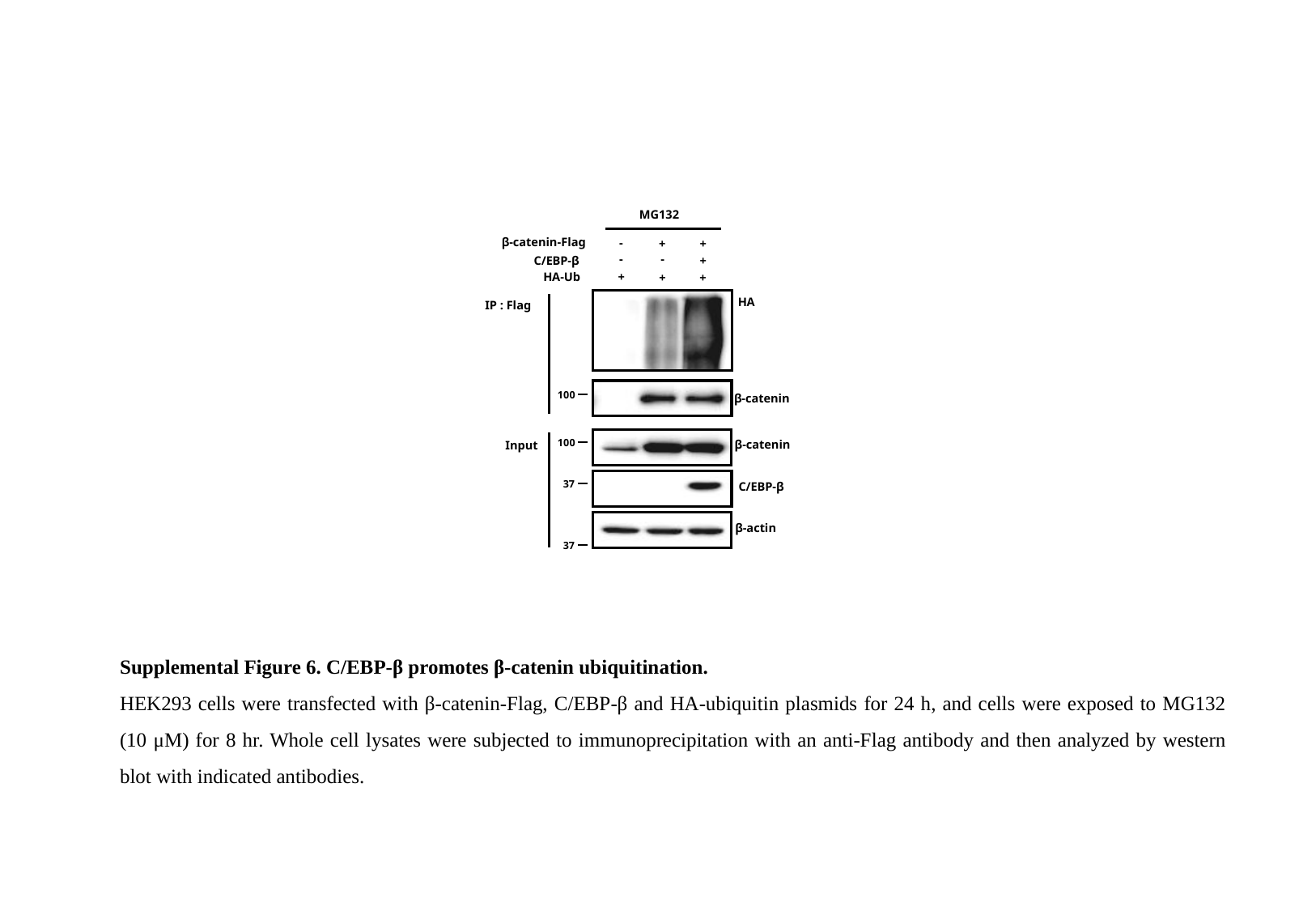

MG132
β-catenin-Flag
-
+
+
-
-
+
C/EBP-β
+
HA-Ub
+
+
HA
IP : Flag
β-catenin
100
β-catenin
Input
37
C/EBP-β
β-actin
37
100
Supplemental Figure 6. C/EBP-β promotes β-catenin ubiquitination.
HEK293 cells were transfected with β-catenin-Flag, C/EBP-β and HA-ubiquitin plasmids for 24 h, and cells were exposed to MG132 (10 μM) for 8 hr. Whole cell lysates were subjected to immunoprecipitation with an anti-Flag antibody and then analyzed by western blot with indicated antibodies.

## Slide 7
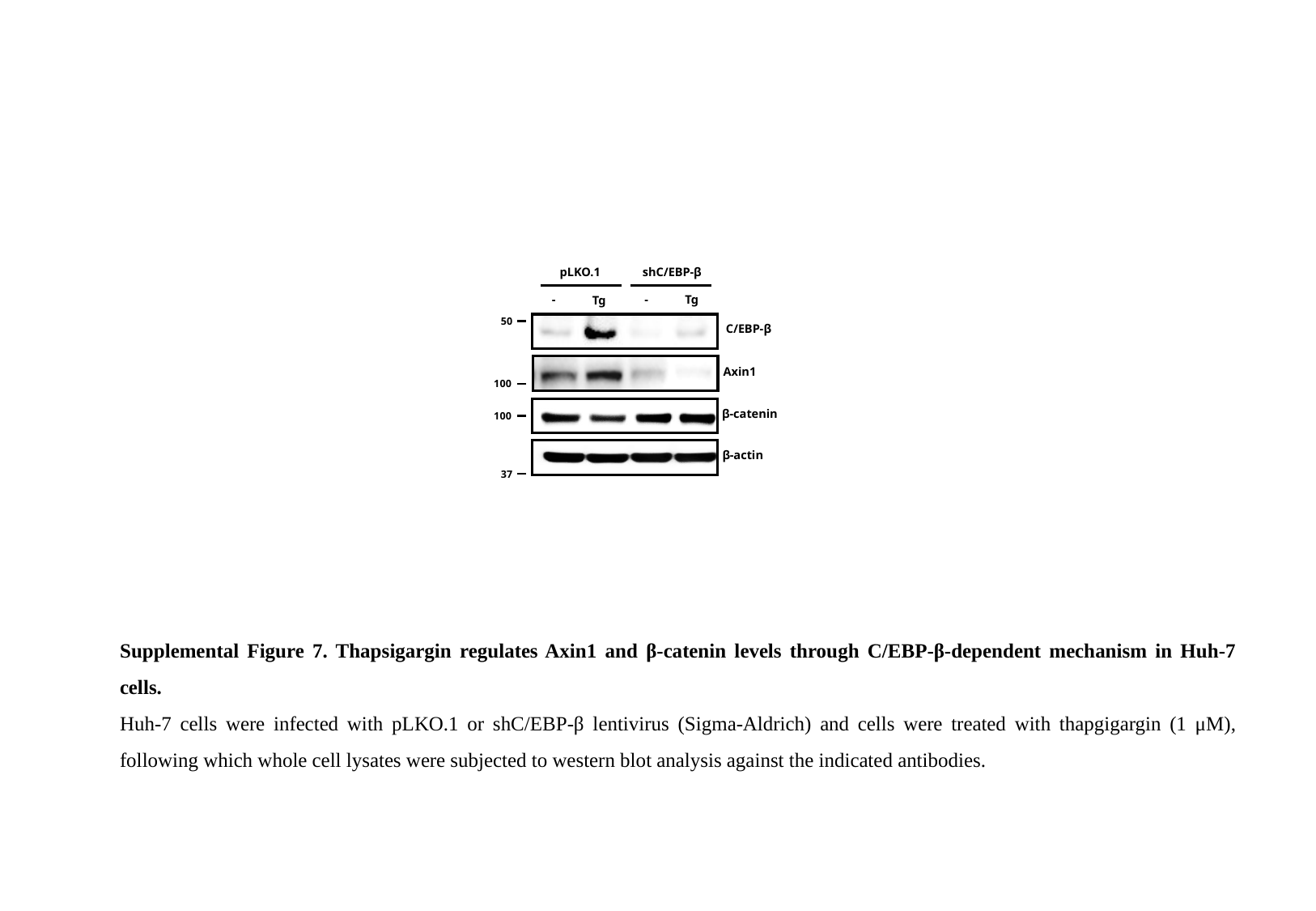

pLKO.1
shC/EBP-β
-
-
Tg
Tg
50
C/EBP-β
Axin1
100
β-catenin
100
β-actin
37
Supplemental Figure 7. Thapsigargin regulates Axin1 and β-catenin levels through C/EBP-β-dependent mechanism in Huh-7 cells.
Huh-7 cells were infected with pLKO.1 or shC/EBP-β lentivirus (Sigma-Aldrich) and cells were treated with thapgigargin (1 μM), following which whole cell lysates were subjected to western blot analysis against the indicated antibodies.
